# Subgenotype VII.1.1 Newcastle Disease Virus Evolution and Spread in the Russian Federation in 2019–2023

**DOI:** 10.3390/v17101319

**Published:** 2025-09-29

**Authors:** Nelly A. Guseva, Sergey N. Kolosov, Nikolay G. Zinyakov, Anton A. Kozlov, Lydia O. Shcherbakova, Irina A. Chvala, Artem V. Andriyasov, Renfu Yin, Dmitry B. Andreychuk, Ilya A. Chvala

**Affiliations:** 1 FGBI “Federal Centre for Animal Health” (FGBI “ARRIAH”), Vladimir 600901, Russia; 2 State Key Laboratory for Diagnosis and Treatment of Severe Zoonotic Infectious Diseases, Key Laboratory of Zoonosis Research, Ministry of Education, Department of Preventive Veterinary Medicine, College of Veterinary Medicine, Jilin University, Changchun 130062, China

**Keywords:** Newcastle disease viruses (NDV), Orthoavulavirus javaense, subgenotype VII.1.1, phylogenetic and evolutionary analysis

## Abstract

Between 2019 and 2023, 163 cases of subgenotype VII.1.1 Newcastle disease virus infection were registered in backyard poultry in the Russian Federation within the framework of epizootiological monitoring. Subgenotype VII.1.1 Newcastle disease virus was reported in a total of 18 different subjects of the Russian Federation. Most of the Newcastle disease outbreaks caused by the viruses of this subgenotype occurred in the autumn and winter period (60%). Further tests allowed for the determination of complete F and HN gene nucleotide sequences for 40 isolates. The results were used to perform the Bayesian analysis of F gene sequences with BEAST v.1.10.4 software. The obtained nucleotide substitution accumulation rates were practically non-dependent on the selected nucleotide substitution model and varied appreciably depending on the applied molecular clock model (0.0018 and 0.002 site-1year-1). The conducted study established that the formation of the ‘Russian’ NDV isolates of subgenotype VII.1.1 followed several stages. In the early 2000s, ancestral viruses belonging to subgenotype VII-d were detected in the Middle East and Eastern Europe. From these, through intermediate forms identified in Iraq around 2007–2008, a group designated as subgenotype VII-L emerged. This group gave rise to two sister clades: the Iranian subgenotype VII-L and the cluster of isolates from Russia and Poland, whose immediate common ancestor likely existed around 2015–2016, probably in Asia.

## 1. Introduction

Newcastle disease (ND) is a highly contagious viral disease of birds, according to the World Organization for Animal Health (WOAH). Newcastle disease virus (NDV), or Orthoavulavirus javaense, is a member of the genus Orthoavulavirus, the family Paramyxoviridae (ICTV) [1].

NDV is characterized by high genetic diversity. In accordance with the classification proposed by Dimitrov et al. (2019) [2], all NDV isolates are divided into 2 classes—class 1 and class 2, which, in its turn, is subdivided into 21 genotypes (I-XXI). In the past 20 years, the ND outbreaks caused by class 2 genotype VII NDV have been posing the greatest threat to the countries of Europe, Asia, and Africa. This genotype includes subgenotypes VII.1.1, 1.2 and 2 [2]. Most common are subgenotypes VII.1.1 and VII.2. Since the late 1990s, subgenotype VII.1.1 has been detected in China [3,4], Iran [5,6], Taiwan [7], Afghanistan [8], Saudi Arabia [9], Iraq [10], Egypt [11,12,13], Indonesia [14], as well as in Japan [15], Vietnam [16], and Eastern Europe [17]. Subgenotype VII.2 was initially detected in the countries of Southeast Asia (Cambodia, Indonesia, Malaysia) in 2007-2014 [18] and West Africa [19], from where it spread to Mozambique [20], Botswana [21], and Tanzania [22], as well as to the countries of Europe and Asia: Israel, Jordan [23], China [20], Pakistan [24], Oman [25], India [26], Bulgaria, Georgia, and Belgium [17,27].

At different times, subgenotypes VII.1.1 (2000–2010) [28] and VII.2 (2013, 2016–2017) [29,30] were detected in Russia.

Since 2019, all ND outbreaks in the Russian Federation have been caused by NDV isolates of subgenotype VII.1.1 (according to the classification by Dimitrov et al. (2019) [2] or subgenotype VII-L (proposed by Sabouri et al. (2018) [5], according to the classification by Diel et al. (2012) [31]). The viruses of this subgenotype were detected in different regions of the country in 2019–2023. Initially, subgenotype VII-L viruses were described by Iranian researchers in 2017 [6,32], who showed their similarity to and close phylogenetic relationship with the previously known subgenotype VII-d. Subsequently, the authors [33,34] increased the number of the studied isolates of this group, showing their distribution in almost all provinces of Iran.

This paper presents the molecular genetics and evolutionary analysis of subgenotype VII.1.1 NDV isolates detected in Russia in 2019–2023. In addition, the obtained sequences were subjected to Bayesian analysis using BEAST software to determine substitution accumulation rates and the time of the main phylogenetic events.

## 2. Materials and Methods

### 2.1. Sampling

In the period from January 2019 to December 2023, the samples of biological material (internal organs, feces, oropharyngeal, and cloacal swabs) from dead poultry and poultry with clinical signs of Newcastle disease were submitted to the National Reference Laboratory for Avian Influenza and Newcastle Disease of the WOAH (Federal State-Financed Institution “Federal Centre for Animal Health” (FGBI “ARRIAH”), Yur′evets, Vladimir, Russia). The samples were collected in the backyards by the specialists of the Territorial Administrations of the Federal Service for Veterinary and Phytosanitary Surveillance (Rosselkhoznadzor) and the Veterinary Departments of the Russian Federation Subjects.

### 2.2. Sample Preparation

The test material (samples of internal organs, tracheal and cloacal flushes, fecal samples) was crushed (if necessary) using sterile scissors and placed in a plastic container for biomaterial. The resulting mass was weighed and diluted with a sterile phosphate buffer solution to obtain a 10–20% suspension. Next, the suspension was transferred to 1.5 mL and 2 mL tubes and centrifuged at 1000–2000× *g* for 15 min. The obtained suspensions of organs were used for RNA extraction and virus isolation.

### 2.3. Virus Isolation

Subgenotype VII.1.1 NDV isolates were detected with RT-PCR in 163 samples from backyard poultry. Each sample was inoculated into 9–11-day-old specific-pathogen-free (SPF) embryonated chicken eggs. The collected allantoic fluid was tested with hemagglutination (HA) assay in accordance with the recommendations of WOAH.

### 2.4. RNA Extraction

RNA extraction from the allantoic fluid and pathological material was carried out using RNeasy Mini Kits (Qiagen, Hilden, Germany) in accordance with the manufacturer’s instructions.

### 2.5. Real-Time Reverse Transcription-Polymerase Chain Reaction (Real-Time RT-PCR)

One-stage real-time RT-PCR for matrix gene for the primary screening of the samples was carried out using the reagent kits Promega AMV Reverse Transcriptase and DNA Polymerase (Promega, Madison, WI, USA), Qiagen OneStep RT-PCR Kit (Qiagen, Hilden, Germany), a deoxyribonucleotide triphosphate mixture (Thermo Scientific, Waltham, Massachusetts, USA), solutions of forward (M4100F 5′-AGT-GAT-GTG-CTC-GGA-CCT-TC-3′) and reverse (M4220R 5′-ATC-GTT-TAC-GGA-GAG-GAG-TCC-3′) primers and fluorescent probe (M4169 5′-(FAM)TTC-TCT-AGC-AGT-GGG-ACA-GCC-TGC (RTQ1)-3′) [35]. Temperature and time conditions for real-time RT-PCR were as follows: reverse transcription—30 min at 50 °C, polymerase activation—10 min at 95 °C, then 40 PCR cycles, each consisting of denaturation—10 s at 95 °C, annealing of primers—35 s at 55 °C, and elongation—10 s at 72 °C.

### 2.6. Reverse Transcription (RT) and Polymerase Chain Reaction (PCR) for Determination of Complete F and HN Gene Sequences

Reverse transcription for cDNA synthesis was carried out using Maxima H Minus Reverse Transcriptase (Thermo Scientific, Waltham, MA, USA), RiboLock RNase Inhibitor (Thermo Scientific, Waltham, MA, USA), a phosphate solution (dNTP) (Thermo Scientific, Waltham, MA, USA), the solution of forward primer VII5 5′-ACCAAACAGAGAATCYGTGAG-3′ and nuclease-free water. Primary PCR (PCR-I) and nested PCR for determination of fusion (F) and heamagglutinin-neuraminidase (HN) gene sequences were carried out using the reagent kits Promega DNA Polymerase (Promega, Madison, WI, USA), DreamTaq DNA Polymerase (Thermo Scientific, Waltham, MA, USA), nuclease-free water, a phosphate solution (dNTP) (Thermo Scientific, USA) and solutions of forward and reverse primers (Table S1).

Temperature and time conditions for primary PCR were as follows: polymerase activation—3 min at 95 °C, then 40 PCR cycles, each consisting of denaturation—20 s at 95 °C, annealing of primers—30 s at 55 °C and elongation—4 min 20 s at 72 °C, followed by additional elongation—10 min at 72 °C. Temperature and time conditions for nested PCR were as follows: polymerase activation—2 min at 95 °C, then 30 PCR cycles, each consisting of denaturation—20 s at 95 °C, annealing of primers—30 s at 55 °C, and elongation—40 s at 72 °C.

### 2.7. Sequencing

The F and HN gene nucleotide sequences of subgenotype VII.1.1 NDV were determined using an automated ABI Prism 3130xl sequencer and BigDye Terminator Cycle Sequencing kits (Applied Biosystems, Waltham, MA, USA), according to the manufacturer’s instructions. The obtained sequences were edited with BioEdit version 7.2 software, as well as submitted to GenBank (Table 1).

### 2.8. Alignment and Editing of Nucleotide Sequences

The alignment and editing of the nucleotide sequences were carried out using ClustalW and BioEdit version 7.2 software. The phylogenetic analysis was carried out with the neighbor-joining method using the HKY model (Kumar S, Stecher G, Li M et al. (2018)) [36]. The robustness of phylogeny was assessed by 500 bootstraps. Discrete gamma distribution was used to model evolutionary rate differences among sites. The analysis was carried out with MEGA X (10.2.6) software.

### 2.9. Dataset for Phylogenetic and Evolutionary Analysis

Two variants of nucleotide sequence analysis were performed: analysis of a fragment of the F gene of NDV isolates of genotype VII identified in poultry in the Russian Federation since 2003, and analysis of the complete nucleotide sequences of the F and HN isolates identified in the Russian Federation in 2019–2023.

For the phylogenetic analysis, a proprietary database of F gene sequences of NDV genotype VII isolates was used, along with sequences from the dataset “NDV_class_II_pilot_128_May_09_2022” [2], and sequences publicly available in GenBank. Neighbor-joining methods were used to construct phylogenetic trees. Neighbor-joining trees (Maximum Composite Likelihood model) were constructed using MEGA X (10.2.6) with 500 bootstrap replicates [2].

The dataset for the evolutionary analysis included 78 F gene sequences of subgenotype VII.1.1 NDV isolates: 40 sequences obtained during this study, 4 sequences from Iraq, and 34 sequences from Iran described above.

### 2.10. Evolutionary Analysis of Nucleotide Sequences Using BEAST and BEAUTI Software

To determine the genetic change rates of the isolates and the time of emergence of the Russian branch of subgenotype VII.1.1, the Bayesian analysis of F gene sequences was performed using BEAST (Bayesian Evolutionary Analysis Sampling Trees) v.1.10.4 software. A set of 40 complete F gene sequences of the Russian isolates and Iranian Beh strain (MF417546.1) was analyzed to root the constructed tree. The Russian isolates were grouped into a special group to estimate the time to the most recent common ancestor (TMRCA). GTR and HKY nucleotide substitution models and gamma distribution of substitution rates (five categories), empirical nucleotide frequency, codon splitting into positions 1, 2, and 3, and strict and uncorrelated relaxed molecular clocks were used. Other parameters of the model were set as default. To estimate the parameters of the model, the Monte Carlo Markov Chain was set to 50 million iterations, so that the effective sample size for each parameter was at least 200. To evaluate the parameter estimates from BEAST, Tracer 1.7.2. Software was used (Rambaut et al., 2018) [37].

### 2.11. Mapping

Maps were created using free Map Chart tools (https://www.mapchart.net/index.html, accessed on 9 September 2025).

## 3. Results

### 3.1. Sampling

**Figure 1:**
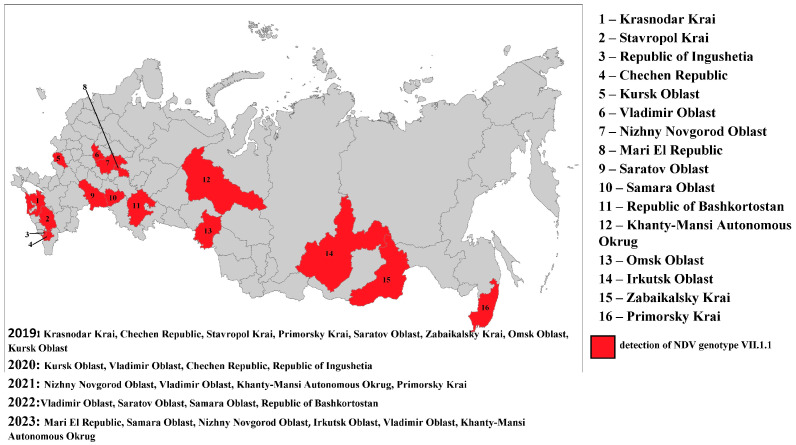
The Russian Federation regions in which subgenotype VII.1.1 NDV isolates were detected in 2019–2023.

In the period from 2019 to 2023, 28,776 samples of biological materials were tested for Newcastle disease virus with real-time RT-PCR. During the study, subgenotype VII.1.1 NDV isolates were detected with real-time RT-PCR and RT-PCR in 36 samples in 2019, in 34 samples in 2020, in 8 samples in 2021, in 51 samples in 2022, and in 34 samples in 2023 (in total, 163 samples). The Russian Federation regions in which subgenotype VII.1.1 NDV isolates were detected in 2019–2023 are shown in Figure 1.

In 2019–2023, subgenotype VII.1.1 viruses were detected in samples collected from backyard poultry. Most of the ND outbreaks caused by the viruses of the said subgenotype occurred in the autumn and winter period (60%). In the Russian Federation, subgenotype VII.1.1 NDV was first detected in the Krasnodar Krai in January 2019. The virus was detected in internal organ samples from non-vaccinated backyard chickens. Then the virus was again isolated from backyard poultry in two other regions in the south of the Russian Federation: in the Chechen Republic in March and in the Stavropol Krai in April. In May, subgenotype VII.1.1 NDV was unexpectedly detected in the Primorsky Krai. The virus was detected in internal organ samples from non-vaccinated chickens. Subsequently, in 2019–2023, the outbreaks of the disease were reported mainly in the autumn and winter period in different geographical locations of the country.

During the work performed, complete F and HN gene nucleotide sequences were determined for 40 isolates. The information on the viruses studied within this work is presented in Table 1.

### 3.2. Phylogenetic Analysis

To detect closely related NDV isolates that circulated prior to 2019, a phylogenetic analysis of the F gene fragment of isolates identified since 2003 was performed.

A total of 54 nucleotide sequences of the F gene fragment of NDV isolates belonging exclusively to genotype VII were selected from the database of the FGBI “ARRIAH”. For the analysis of the F gene fragment, the most closely related nucleotide sequences from GenBank and the dataset “NDV_class_II_pilot_128_May_09_2022” [2] were used.

**Figure 2:**
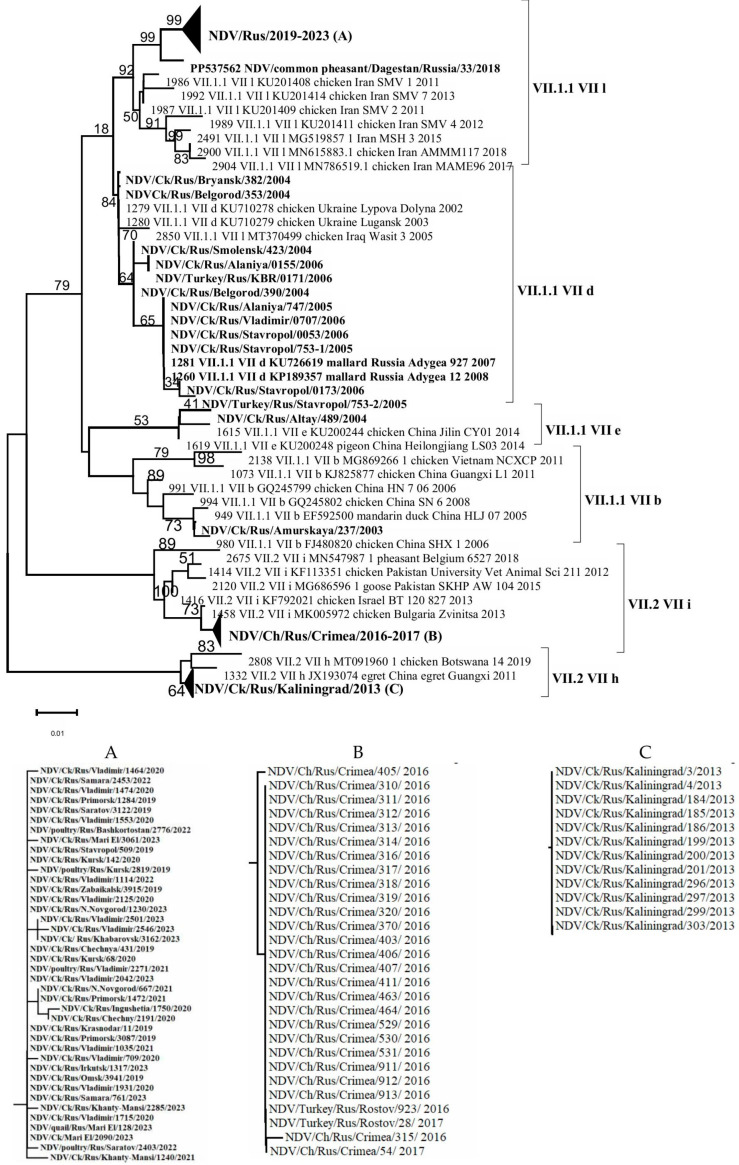
Phylogenetic tree of NDV isolates of genotype VII (partial F gene ORF nucleotides 212-494). (**A**–**C**) Compressed and expanded trees.

Alignment-based analysis of 94 (54 + 40) F gene fragment sequences enabled the identification of genetic groups/subgenotypes of NDVisolates detected in the Russian Federation during the period 2003–2023. It was shown that 56 isolates belonged to subgenotype VII.1.1, which represents a large and genetically diverse group of NDV. This subgenotype includes viruses that caused the fourth ND panzootic and were previously classified under subgenotypes VII-b, VII-d, VII-e, VII-j, and VII-L [2,31]. In contrast, 28 isolates were assigned to subgenotype VII.2 (VII-i, VII-h) [1,30] (Figure 2).

As a result of the genetic analysis of NDV isolates collected in the Russian Federation, it was established that isolates belonging to subgenotypes VII-b, VII-d, and VII-e were detected in 2003–2004; in 2005–2006, only subgenotype VII-d was identified; in 2013, only VII-h; in 2016–2017, only VII-i; and in 2019–2023, exclusively subgenotype VII-L. The relatively low number of NDV isolates of subgenotypes VII-h and VII-i detected in 2013 and 2016–2017, respectively, may be attributed to a decrease in the volume of surveillance studies during that period, which likely affected the objectivity of the epizootic picture. On the other hand, a relatively extensive group of closely related isolates from 2019 to 2023 was identified in backyard poultry farms located in various regions of the Russian Federation during comprehensive nationwide surveillance efforts conducted in that period (Figure 1).

It is noteworthy that the VII-d subgenotype isolates detected in 2004–2007 were genetically close to the virulent NDV isolates of subgenotype VII-L identified in 2019–2023. Subgenotype VII-d is globally distributed and has been one of the most widespread circulating subgenotypes among poultry and wild birds since the early 21st century [28]. Phylogenetic analysis of the full coding sequence of the F gene from isolates identified in Bulgaria and Ukraine, conducted by Dimitrov et al. (2016) [28], showed that VII-d viruses replaced strains that had been circulating in Bulgaria prior to 2002. A similar pattern appears to have occurred with NDV isolates in the Russian Federation, and our findings support the conclusions of Dimitrov and colleagues [28].

To enhance the precision of the genetic analysis, complete nucleotide sequences of the F and HN genes were obtained for NDV isolates collected during 2019–2023. These data enabled a comprehensive comparative analysis between the Russian isolates and reference virus sequences available in the ‘NDV_class_II_pilot_128_May_09_2022’ dataset [2] as well as in the GenBank database (ncbi.nlm.nih.gov).

**Figure 3:**
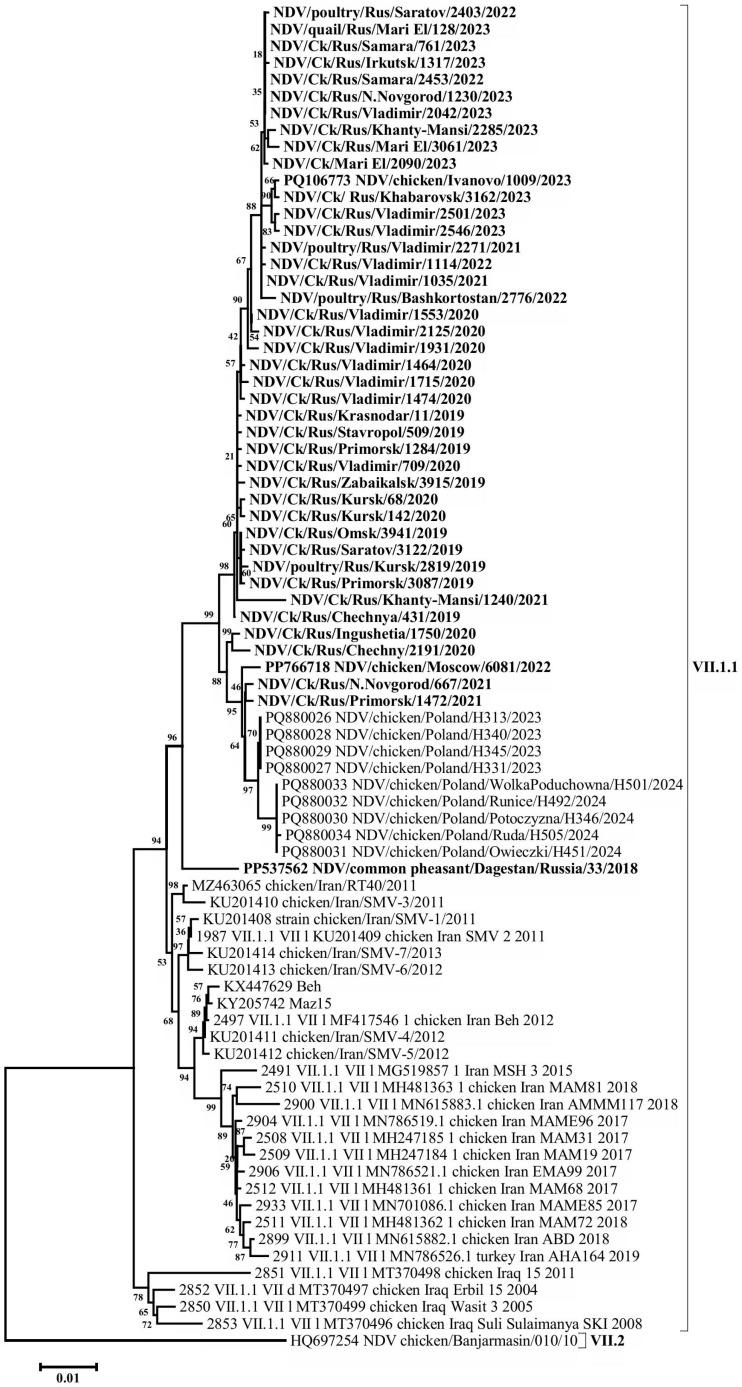
Phylogenetic tree of NDV isolates of genetic group VII-L (subgenotype VII.1.1) (F gene ORF nucleotides 1-1661).

**Figure 4:**
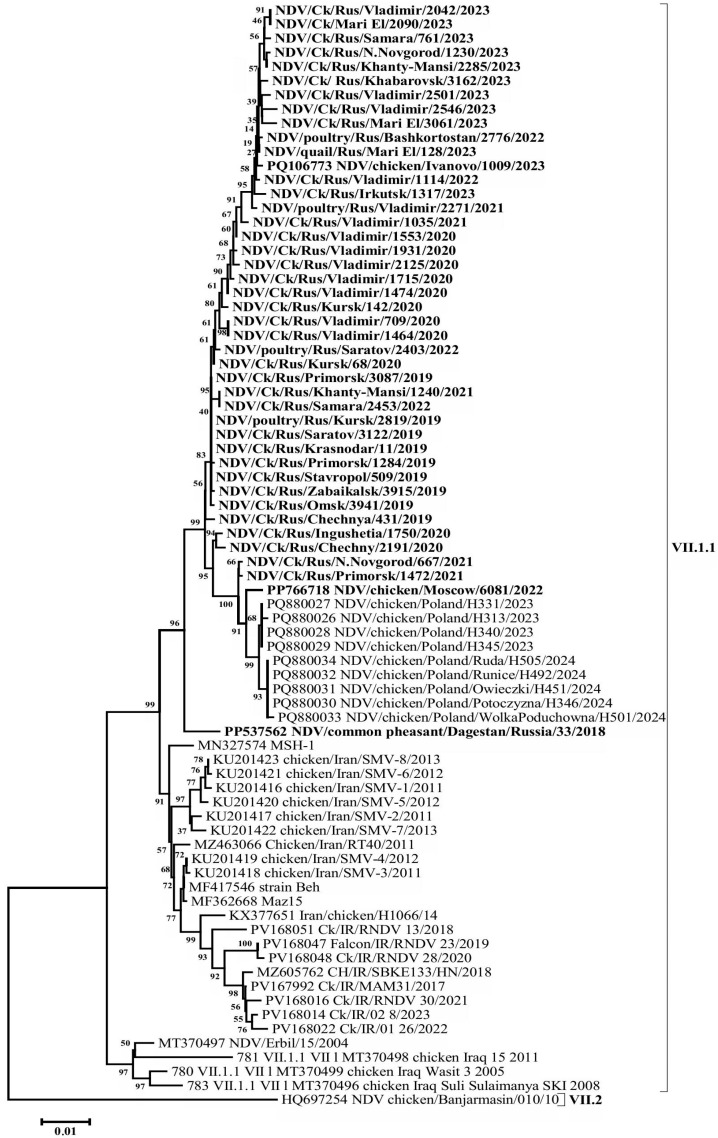
Phylogenetic tree of NDV isolates of genetic group VII-L (subgenotype VII.1.1) (HN gene ORF nucleotides 1-1713).

A BLAST (v. 2.16.0 software) search for the most similar sequences revealed that the closest matches to the Russian isolates from 2019 to 2023 were a group of isolates detected in Poland in 2023–2024, showing 99.58–99.16% nucleotide identity in the F gene. In comparison, VII-L subgenotype isolates from Iran, collected between 2011 and 2015, exhibited up to 97.95% identity. A high level of similarity (96.33–97.05%) was also observed when compared with the isolates of the said subgenotype detected in Iran in 2017–2020 and with subgenotype VII-d isolates detected in Iraq in 2005 and 2008 (Figure 3, Figure 4 and Figure S1).

Phylogenetic relationships for this group of isolates were determined based on the complete nucleotide sequences of the F and HN genes. The phylogenetic trees constructed for both genes displayed a high degree of congruence, indicating the absence of recombination events—a characteristic feature of the NDV genome. The evolutionary pathway can also be clearly traced. The phylogenetic group of VII-L subgenotype isolates appears to have originated from an ancestral group of VII-d subgenotype isolates that entered the Middle East and Eastern Europe from China in the early 2000s [28], likely via intermediate forms from Iraq. The Russian isolates, along with the closely related Polish isolates (which likely descended from them), and the Iranian VII-L isolates appear to represent sister clades that diverged shortly after the emergence of this subgenotype.

The group of VII.1.1 subgenotype isolates detected in the Russian Federation has a number of specific nucleotide substitutions that place them into a separate genetic lineage within group VII-L. In particular, F gene open reading frame has the following substitutions common to all the Russian Federation isolates: 114A->T, 152A->G, 210C->T, 294C->T, 348T->C, 441T->C, 474T->C, 480A->G, 504C->T, 564C->A, 651G->A, 748T->C, 810T->C, 834A->G, 906T->C, 948A->G, 1008A->C, 1093C->T, 1131C->G, 1217C->A, 1281A->G, 1401T->C, 1455C->A, 1490G->A, 1497T->C (in total, 25 nucleotide substitutions). Most of these substitutions are synonymous transitions (17 substitutions), but synonymous transversions are also present (3 substitutions). At the same time, 5 out of 25 substitutions in the group of the Russian isolates are non-synonymous: 152A->G (N->S), 564C->A (D->E), 1217C->A (G->S), 1455C->A (N->K), 1490G->A (S->N).

The analysis of HN gene ORF nucleotide sequence also revealed substitutions common to all the Russian Federation isolates: 30G->A, 82A->G, 103A->G, 456T->C, 470A->G, 507A->G, 510C->T, 552C->T, 615C->T, 618G->A, 861C->T, 903G->A, 1026C->T, 1066A->G, 1161A->G, 1278A->G, 1450C->T, 1453A->C, 1470G->A, 1490C->A, 1503C->T, 1518C->T, 1530T->C, 1623T->C, 1629T->C, 1684C->T (in total, 26 nucleotide substitutions). As in the case of the F gene, most of these substitutions are synonymous transitions (21 substitutions). It should be noted that 5 out of 26 substitutions are non-synonymous: 82A->G (T->A), 103A->G (M->V), 470A->G (Q->R), 1066A->G (K->E), 1490C->A (A->E).

### 3.3. Evolutionary Analysis of Nucleotide Sequence Variability of NDVisolates

As part of the study, the Bayesian analysis of the obtained F gene sequences was performed using BEAST software to determine substitution accumulation rates and the time of the main phylogenetic events.

The point mutation accumulation rate in the group of Russian subgenotype VII.1.1 isolates (*n* = 40) for five years (2019–2023) was determined using BEAST software for GTR and HKY nucleotide substitution models based on the strict and uncorrelated relaxed molecular clock models. The obtained nucleotide substitution accumulation rates were practically non-dependent on the selected nucleotide substitution model and varied appreciably depending on the applied molecular clock model (0.0018 and 0.002 site-1year-1), which was also observed for NDV by other authors for NDV [38] (Table 2).

## 4. Discussion

Isolates of subgenotype VII.1.1 (VII-L) first appeared in the Russian Federation in 2019 and began to spread rapidly throughout the country. Isolates of the same genetic group were detected in Poland in 2023–2024. The high level of genetic similarity between the Russian and Polish isolates suggests that they represent a new wave of the extensive epizootic process in Eastern Europe described by Dimitrov et al. [28].

The analysis of identified single nucleotide substitutions indicates a distinct evolutionary pathway of the potential ancestor of the Russian isolate group over a certain period of time, likely around five years. The spread of NDV subgenotype VII.1.1 across the Russian Federation within a single year was rapid and unpredictable. In 2019, outbreaks were recorded in geographically distant regions, ranging from the Caucasus to the Far East. The first three outbreaks occurred in the southern regions of the country, followed by detections in the Far Eastern region, and subsequently in the central part of the country. Comparison of the nucleotide sequences of the detected viruses reveals their close genetic relatedness and descent from a common ancestor. The early branching point of the Russian and Iranian isolate groups, along with the absence of intermediate forms for the initial isolates, suggests the likely existence of a common ancestor in an unknown region of Asia.

The hypothesis of the Middle East as the original source of subgenotype VII.1.1 viruses is supported by the estimated dating of the root node in the phylogenetic tree. For our dataset, the root node dating across different models averaged around 2008–2009, preceding the earliest published isolates from Iran dated to 2010. This indicates an early divergence of the future ‘Russian’ branch of subgenotype VII.1.1. At the same time, the estimated date of the most recent common ancestor of the Russian isolate group (2016–2017) closely corresponds to the detection date of the earliest isolate identified by researchers from Novosibirsk (NDV/common pheasant/Dagestan/Russia/33/2018) in 2018. Together with the phylogenetic data, this supports a virtually monophyletic origin of the group.

The widespread and rapid dissemination of NDV subgenotype VII.1.1 viruses across the Russian Federation, and subsequently Poland, raises several questions regarding the factors facilitating the virus’s spread over considerable distances. The involvement of migratory birds in the transmission of NDV among domestic poultry has been repeatedly discussed in previous studies [39,40,41].

The list of bird species in which NDV genotype VII has been detected is extensive, with chickens being the most frequently studied host. Besides chickens, the list includes turkeys, pheasants, and quail, as well as waterfowl such as mallard, mandarin duck, sheldrake duck, muscovy duck, goose, bean goose, white-fronted goose, common teal, and black swan; near-water birds like crested ibis and egret; raptors including rough-legged buzzard, long-eared owl, Japanese sparrowhawk, sparrowhawk; as well as penguins, parrots, parakeets, peacocks, and ostriches [42,43,44].

NDV genotype VII has also been detected in pigeons [28,34,45]. Pigeons are considered effective carriers and transmitters of subgenotype VII-d viruses to commercially raised broiler chickens kept in open poultry houses [46]. Nevertheless, we did not detect genotype VII viruses in pigeons. Our identification of NDV in pigeons during 2019–2023 revealed only genotypes VI and XXI. Access to domestic poultry in small private farms (SPFs) is not limited to pigeons alone. It may be necessary to broaden the range of bird species utilizing SPFs as a feeding base to identify those involved in the NDV transmission chain.

The direct or indirect influence of humans on the spread of NDV cannot be excluded. The role of anthropogenic factors in the dissemination of NDV related to trade and transport of industrial poultry is supported by several studies from Iran [33,35]. Understanding how humans contribute to the spread of Newcastle disease virus (via trade and/or illegal movement of birds and poultry products) could significantly reduce the economic impact of the disease.

The conducted study established that the formation of the ‘Russian’ NDV isolates of subgenotype VII.1.1 followed several stages. In the early 2000s, ancestral viruses belonging to subgenotype VII-d were detected in the Middle East and Eastern Europe. From these, through intermediate forms identified in Iraq around 2007–2008, a group designated as subgenotype VII-L emerged. This group gave rise to two sister clades: the Iranian subgenotype VII-L and the cluster of isolates from Russia and Poland, whose immediate common ancestor likely existed around 2015–2016, probably in Asia.

## Figures and Tables

**Table 1:** Information on subgenotype VII.1.1 NDV isolates detected in the Russian Federation in 2019–2023.

No.	Isolate	Sampling Region	Species	Sampling Date	Accession F/HN
1	NDV/Ck/Rus/Krasnodar/207/2019	Krasnodar Krai	Chicken	29 January 2019	PV490921 PV490946
2	NDV/Ck/Rus/Chechnya/819/2019	Chechen Republic	Chicken	13 March 2019	PV490922 PV490947
3	NDV/Ck/Rus/Stavropol/923/2019	Stavropol Krai	Chicken	18 April 2019	PV490923 PV490948
4	NDV/Ck/Rus/Primorsk/1134/2019	Primorsky Krai	Chicken	6 May 2019	PV490924 PQ641107
5	NDV/Ck/Rus/Saratov/2017/2019	Saratov Oblast	Chicken	1 August 2019	PV490925 PQ641108
6	NDV/Ck/Rus/Zabaikalsk/2346/2019	Zabaikalsky Krai	Chicken	4 September 2019	PV490926 PV490949
7	NDV/Ck/Rus/Omsk/2366/2019	Omsk Oblast	Chicken	October 2019	PV490927 PV490950
8	NDV/poultry/Rus/Kursk/2819/2019	Kursk Oblast	Chicken	November 2019	PV490928 PV490951
9	NDV/Ck/Rus/Primorsk/3087/2019	Primorsky Krai	Chicken	10 December 2019	PV490929 PV490952
10	NDV/Ck/Rus/Kursk/68/2020	Kursk Oblast	Chicken	January 2020	PV490930 PQ641109
11	NDV/Ck/Rus/Kursk/142/2020	Kursk Oblast	Chicken	January 2020	PV490931 PQ641110
12	NDV/Ck/Rus/Vladimir/709/2020	Vladimir Oblast	Chicken	30 April 2020	PV490932 PV490953
13	NDV/Ck/Rus/Vladimir/1464/2020	Vladimir Oblast	Chicken	4 September 2020	PV490933 PQ641111
14	NDV/Ck/Rus/Vladimir/1474/2020	Vladimir Oblast	Chicken	7 September 2020	PV490934 PQ641112
15	NDV/Ck/Rus/Vladimir/1553/2020	Vladimir Oblast	Chicken	14 September 2020	PV490935 PQ641113
16	NDV/Ck/Rus/Vladimir/1715/2020	Vladimir Oblast	Chicken	7 October 2020	PV490936 PQ641114
17	NDV/Ck/Rus/Ingushetia/1750/2020	Republic of Ingushetia	Chicken	13 August 2020	PV490937 PV490954
18	NDV/Ck/Rus/Vladimir/1931/2020	Vladimir Oblast	Chicken	5 November 2020	PV490938 PQ641115
19	NDV/Ck/Rus/Vladimir/2125/2020	Vladimir Oblast	Chicken	1 December 2020	PV490939 PQ641116
20	NDV/Ck/Rus/Chechny/2191/2020	Chechen Republic	Chicken	7 December 2020	PV490940 PQ641117
21	NDV/Ck/Rus/N.Novgorod/667/2021	Nizhny Novgorod Oblast	Chicken	1 May 2021	PV490941 PQ641118
22	NDV/Ck/Rus/Vladimir/1035/2021	Vladimir Oblast	Chicken	20 July 2021	PV490942 PQ641119
23	NDV/Ck/Rus/Khanty-Mansi/1240/2021	Khanty-Mansi Autonomous Okrug	Chicken	9 August 2021	PV490943 PV490955
24	NDV/Ck/Rus/Primorsk/1472/2021	Primorsky Krai	Chicken	6 September 2021	PV490944 PQ641120
25	NDV/poultry/Rus/Vladimir/2271/2021	Vladimir Oblast	Chicken	28 December 2021	PV490945 PV490956
26	NDV/Ck/Rus/Vladimir/1114/2022	Vladimir Oblast	Chicken	8 June 2022	PQ641093 PQ641121
27	NDV/poultry/Rus/Saratov/2403/2022	Saratov Oblast	Chicken	October 2022	PQ641094 PQ641122
28	NDV/Ck/Rus/Samara/2453/2022	Samara Oblast	Chicken	26 October 2022	PQ641095 PQ641123
29	NDV/poultry/Rus/Bashkortostan/2776/2022	Republic of Bashkortostan	Chicken	5 December 2022	PQ641092
30	NDV/quail/Rus/Mari El/128/2023	Mari El Republic	Quail	25 January 2023	PQ641096 PQ641124
31	NDV/Ck/Rus/Samara/761/2023	Samara Oblast	Chicken	April 2023	PQ641097 PQ641125
32	NDV/Ck/Rus/N.Novgorod/1230/2023	Nizhny Novgorod Oblast	Chicken	May 2023	PQ641098 PQ641126
33	NDV/Ck/Rus/Irkutsk/1317/2023	Irkutsk Oblast	Chicken	24 May 2023	PQ641099 PQ641127
34	NDV/Ck/Rus/Vladimir/2042/2023	Vladimir Oblast	Chicken	15 August 2023	PQ641100 PQ641128
35	NDV/Ck/Mari El/2090/2023	Mari El Republic	Chicken	8 August 2023	PQ641101 PQ641129
36	NDV/Ck/Rus/Khanty-Mansi/2285/2023	Khanty-Mansi Autonomous Okrug	Chicken	23 August 2023	PQ641102 PQ641130
37	NDV/Ck/Rus/Vladimir/2501/2023	Vladimir Oblast	Chicken	October 2023	PQ641103 PQ641131
38	NDV/Ck/Rus/Vladimir/2546/2023	Vladimir Oblast	Chicken	October 2023	PQ641104 PQ641132
39	NDV/Ck/Rus/Mari El/3061/2023	Mari El Republic	Chicken	22 November 2023	PQ641105 PQ641133
40	NDV/Ck/Rus/Khabarovsk/3162/2023	Khabarovsk Krai	Chicken	21 November 2023	PQ641106 PQ641134

**Table 2:** Substitution accumulation rates and dates of main phylogenetic events for Russian subgenotype VII.1.1 isolates.

Summary Statistics	Models Used and Parameter Estimates			
	GTR		HKY	
	Strict Model	Relaxed Model	Strict Model	Relaxed Model
	Substitution Accumulation Rate Per Site Per Year for the RF Isolates			
Mean	1.8 × 10^−3^	2.0 × 10^−3^	1.8 × 10^−3^	2.0 × 10^−3^
Standard deviation of mean	3.4310 × 10^−4^	5.47 × 10^−4^	3.47 × 10^−4^	5.50 × 10^−4^
	Date of tree root node for the group of Iranian and Russian isolates			
Mean	2008.9373	2008.9651	2007.8821	2008.5288
Standard deviation of mean	1.5625	1.5531	2.935	2.2313
	Date of the most recent common ancestor of the group of Russian isolates			
Mean	2016.7133	2016.739	2015.7992	2016.4382
Standard deviation of mean	0.8278	0.8222	1.6926	1.1471

## Data Availability

The article shows the numbers under which the data was deposited in GenBank.

## Supplementary Materials

The following supporting information can be downloaded at: https://www.mdpi.com/article/10.3390/v17101319/s1, Figure S1: Phylogenetic tree of NDV isolates (F gene ORF nucleotides 1-1661); Table S1: The structure of primers for amplification and determination of the primary structure of the F and HN genes of the VNB genotype VII.
