# Supplementary material for: Subgenotype VII.1.1 Newcastle Disease Virus Evolution and Spread in the Russian Federation in 2019–2023
Source: Viruses. 2025 Sep 29;17(10):1319. doi: 10.3390/v17101319 (PMC12567687; doi:10.3390/v17101319)
Supplement: Supplementary file 1 [file viruses-17-01319-s001.zip › Table S1.The structure of primers for amplification and determination of the primary structure of the F and HN genes of the VNB genotype VII.pdf]

Table S1. The structure of primers for amplification and determination of the primary structure of the F and HN genes of the VNB genotype VII

Name	Nucleotide sequence 5′-3′
FVII-287f	CGG-AGY-GTG-AAA-GTC-ATC-AT
FVII-54f	AGA-AAA-AAC-ACG-GGT-AGA-AG
FVII397r	GCA-GCT-GCT-GTT-ATY-TGY-GC
FVII181f	ATC-ATA-RTC-AAR-TTG-CTC-CC
FVII775r	CCR-ATT-AAT-GAR-CTG-AGT-TG
FVII646f	GAR-TTG-ACY-ACA-GTA-TTC-GG
FVII1048r	GAC-ATR-GGG-AAT-GTC-ACT-AT
FVII972f	ACA-AGT-YGG-TTC-YGT-GAT-AG
FVII1327r	GAG-ATR-TTC-TTT-WGA-TAA-GT
FVII1239f	AGA-AGC-TRT-ATC-YCT-GAT-AG
FVII1471f	CAT-TGC-TGA-TTG-ART-TRT-TG
FVII1782r	GGC-TCC-TCT-GRC-CGT-TCT-AC
FHNVII1624f	AAT-ACY-CTY-GAT-CAG-ATG-AG
FHNVII2111f	AGA-TAG-GAT-ATA-YAA-RCA-GG
FHNVII2139r	AGT-AGY-GCC-AGC-GGR-GAT-TC
FHNVII2244f	CCY-GTT-CAT-GAC-CCA-GAY-TA
FHNVII2378f	GGT-TGC-ACT-CGG-ATA-CCC-TC
FHNVII2748r	GGR-TAA-TTT-GCC-ACC-CAA-TC
FHNVII2799r	TAA-ACY-GGG-AAC-CAT-ACA-CG
FHNVII3330f	TTC-GGR-ACR-ATG-CTT-GAT-GA
FHNVII3417r	TTG-GTG-CTG-CTT-GAA-CTY-AC
FHNVII3833r	GCC-CGC-CAT-GTC-CTA-CCC-GT
FHNVII3857r	GTG-CTC-TGC-CCT-YTC-GGG-AC
