# Supplementary figures and images for: Subgenotype VII.1.1 Newcastle Disease Virus Evolution and Spread in the Russian Federation in 2019–2023
Source: Viruses. 2025 Sep 29;17(10):1319. doi: 10.3390/v17101319 (PMC12567687; doi:10.3390/v17101319)

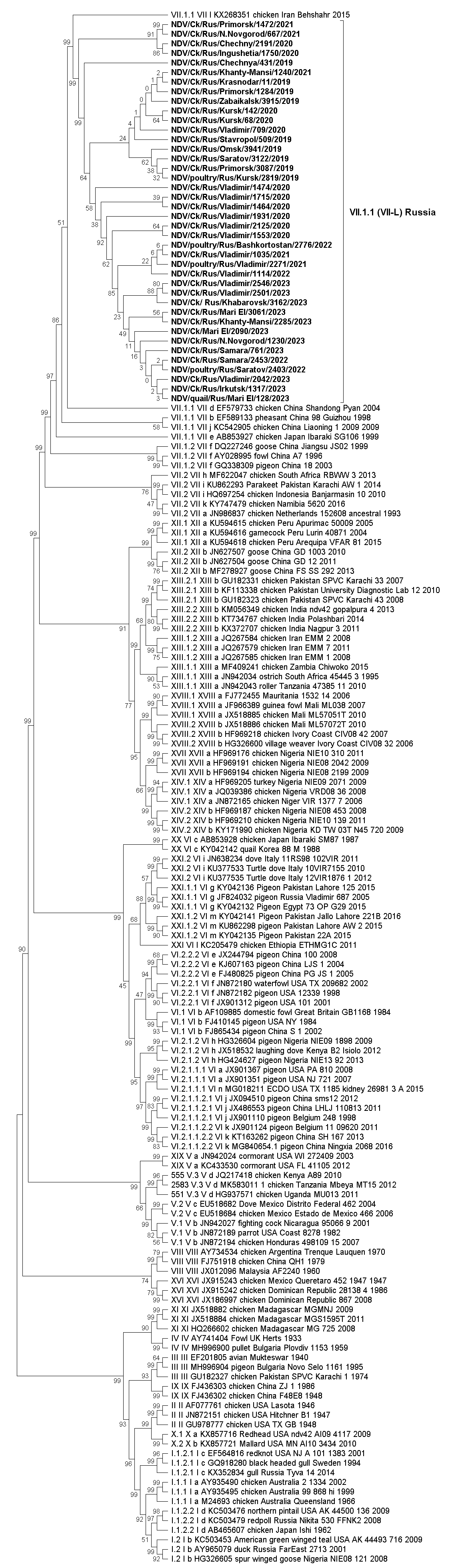

Supplement: Supplementary file 1 [file viruses-17-01319-s001.zip › Figure S1.Phylogenetic tree of NDV isolates (F gene ORF nucleotides 1-1661).tiff]
